# Association of subclass distribution of insulin antibody with glucose control in insulin-treated type 2 diabetes mellitus: a retrospective observational study

**DOI:** 10.3389/fendo.2023.1141414

**Published:** 2023-04-18

**Authors:** Shuang Chen, Heng Chen, Yin Jiang, Xuqin Zheng, Mei Zhang, Tao Yang, Yong Gu

**Affiliations:** Department of Endocrinology, First Affiliated Hospital of Nanjing Medical University, Nanjing, China

**Keywords:** insulin antibody (IA), subclass, type 2 diabetes, glycemic control, retrospective

## Abstract

**Objective:**

To examine the distribution and effects of the subclass of insulin antibodies on glucose control and side events in patients with type 2 diabetes treated with premixed insulin analog.

**Methods:**

A total of 516 patients treated with premixed insulin analog were sequentially enrolled from the First Affiliated Hospital of Nanjing Medical University from June 2016 to August 2020. Subclass-specific insulin antibodies (IAs) (IgG1-4, IgA, IgD, IgE, and IgM) were detected in IA-positive patients by electrochemiluminescence. We analyzed glucose control, serum insulin, and insulin-related events between IA-positive and IA-negative groups, as well as among patients with different IA subclasses.

**Results:**

Overall, 98 of 516 subjects (19.0%) were positive for total IAs after premixed insulin analog therapy; of these participants, 92 had subclass IAs, and IgG-IA was the predominant subclass, followed by IgE-IA. IAs were associated with serum total insulin increase and local injection-site reactions but not glycemic control and hypoglycemia. In the subgroup analysis in patients with IA-positive, the IgE-IA and IA subclass numbers were more associated with increased serum total insulin levels. Additionally, IgE-IA might be correlated more strongly with local responses and weakly with hypoglycemia, while IgM-IA might be correlated more strongly with hypoglycemia.

**Conclusion:**

We concluded that IAs or IA subclasses might be associated with unfavorable events in patients receiving premixed insulin analog therapy, which can be used as an adjunctive monitoring indicator in clinical insulin trials.

## Introduction

Diabetes has been effectively managed with insulin since it was discovered in the 1920s by Banting and Best (1). However, immunological reactions (especially allergic reactions) to insulin have become increasingly common since that time. In their work, Berson et al. (2, 3) found that most insulin-treated patients had insulin-binding immunoglobulins (Igs), which were later identified as polyclonal immunoglobulin G (IgG). Insulin treatment has progressed through the use of animal insulin, recombinant human insulin, and insulin analogs (1, 4), resulting in a remarkable reduction rather than elimination of insulin antibodies. According to Fineberg et al. (5), insulin antibodies (IAs) were still present in 40–60% of insulin-treated diabetics. Although previous studies showed no significant correlation between IAs and glucose control (6, 7), IA-associated cases leading to severe clinical events, including insulin resistance (8), recurrent diabetic ketoacidosis (DKA) (9), or hypoglycemia (10), have continued to be reported. Recently, these events have garnered a resurgence of attention and have been defined as comprising exogenous insulin antibody syndrome (EIAS) (10, 11). Previous studies on the relationship between IAs and glucose control have mainly focused on total insulin antibodies without considering IA subclasses. Moreover, most of these studies were conducted in the 1980s and 1990s (6, 7) and thus should be updated. Indeed, aside from IgG-IA, several other different subtypes of insulin antibodies have been reported (12–15), including immunoglobulin M (IgM), immunoglobulin A (IgA), and immunoglobulin E (IgE). Studies on patients with type 1 diabetes have indicated that different IA subtypes exhibited various predictive effects in research settings (16, 17). Little evidence exists, however, of analysis regarding the association between IA subclasses and glycemic control in insulin-treated patients with type 2 diabetes (T2D). In most clinical trials involving insulin, hypoglycemia and local injection reactions have been the main adverse effects (18, 19); however, there is a lack of markers suggesting or warning of the occurrence of these unfavorable events. We hypothesized that IA subclasses might have a negative impact on glycemic control in clinical settings. Therefore, our study aimed to demonstrate whether IAs or IA subclasses induced by exogenous insulin affected metabolic control and predicted adverse events in Chinese type 2 diabetic patients receiving insulin treatment.

## Methods

### Subjects

Between June 2016 and August 2020, we initially collected 612 consecutive patients receiving premixed insulin analogs (lispro mix 50/50). The inclusion criteria were as follows (1): patients with a diagnosis aged ≥18 years (2); patients with type 2 diabetes diagnosed using WHO diagnostic criteria (3); patients negative for glutamic acid decarboxylase antibody, insulinoma-associated protein 2 antibody, zinc transporter 8 antibody, and insulin autoantibody before receiving insulin therapy (4); patients taking combined oral medication—metformin only (0.5 g, thrice times a day)—while not altering the regimen during the first four months of treatment; and (5) patients with well-documented clinical data and laboratory data (insulin antibody results before and after treatment and IA subclasses of IA-positive patients were required). The exclusion criteria were as follows (1): patients who were IA-positive before insulin therapy; and (2) patients with impaired hepatorenal function, acute diabetic complications, history of steroid use, uncontrolled hypertension, moderate to severe anemia, heart disease including decompensated cardiac insufficiency, unstable angina pectoris, myocardial infarction, active proliferative diabetic retinopathy or other unstable retinopathy, as well as drug abuse or alcohol dependence history. Finally, we included 516 patients receiving lispro mix 50/50. Patients were assigned into the following two groups (1): the IA-negative group, including those who were negative for IAs after insulin administration, comprised of 418 patients (207 males and 211 females) with a median age of 56.7 years, ranging from 29 to 75 years; and (2) the IA-positive group, including those who were positive for IAs after insulin administration, comprised of 98 patients (56 males and 42 females) with a median age of 57.7 years, ranging from 38 to 75 years. The flow chart of our study process is illustrated in **Supplementary Figure 1**.

This project was approved by the ethics committee of the First Affiliated Hospital of Nanjing Medical University (2021-SR-075).

### Clinical characteristics and biochemical measurements

For all included subjects, detailed demographic profiles, clinical characteristics, and laboratory data before and after insulin administration were retrospectively collected, as well as gender, age, diabetic duration, weight, height, blood pressure, and daily insulin dosage (unit/kg per day) data. Glucose concentrations were measured using hexokinase. Serum total cholesterol (TC), triglyceride (TG), low-density lipoprotein cholesterol (LDLC), high-density lipoprotein cholesterol (HDLC), blood urea nitrogen (BUN), creatinine (Cr), alanine aminotransferase (ALT), total bilirubin (TBil), and direct bilirubin (DBil) were measured using an automatic biochemical analyzer (Beckman Coulter AU5800). Serum insulin was analyzed using an electrochemiluminescence (ECL) immunoassay (YHLO iFlash3000). Bio-Rad D-100 high-performance liquid chromatography was used to measure hemoglobin glycated (HbA1c).

### Assays for IAs and their immunoglobulin subclasses

The total IAs assay in our lab was detected using ECL assay, as described in detail in our previous work (20). The sensitivity of the IAs assay was 82.0%, and the specificity was 98.7%. The cut-off index of positivity for IAs was 0.0042, which was determined to represent the 99th percentile of 142 healthy control subjects. IA subclasses were analyzed using the same principles as those used for total IAs. Briefly, a mixture of serum samples with sulfo-tag conjugated proteins (Meso Scale Discovery, R91AO-2) in phosphate-buffered solution (PBS) with 5% bovine serum albumin (BSA)was prepared. Overnight incubation at 4°C with secondary antibodies labeled with biotin against IgG1-4, IgA, IgD, IgM, and IgE (Ab 99775; Invitrogen 05-3540; Ab 86252; Ab 99818; Ab 85864; Ab 224182; Ab 99745; Ab 99807) was performed. Meanwhile, a streptavidin-coated (MesoScale Discovery, L15SA-1) plate with blocker buffer (Meso Scale Discovery, R93AA-1) was incubated under the same conditions. The following day, the mixture of serum and antigen was transferred to the streptavidin plate after it had been washed and incubated at room temperature for one hour; the plate shaker was set at low speed. Following another washing of the plate and the adding of a read buffer, the plate was counted on a plate reader. Using positive and negative control serum samples as internal standards, we generated an index to represent the results. A single assay run was conducted on all samples from each individual.

### Statistical analysis

Continuous variables are expressed as mean ± SD when data are normally distributed or as median (inter-quartile range) when data are not normally distributed. Categorical variables are reported as the numbers (frequency). Differences in clinical characteristics between the groups were analyzed *via* the Student’s *t* test or Mann–Whitney U test for continuous data and the *χ^2^* test for categorical data. For all tests, p values < 0.05 were considered significant with a two-tailed test. Data analysis was carried out using SPSS v25.0 (IBM Co., Armonk, NY, USA), and graphs were generated using PRISM v9.0.0 (GraphPad Software, Inc., La Jolla, CA) and R software (version 4.1.1).

## Results

### Baseline clinical and demographic characteristics of the study population

A total of 516 subjects (253 females and 263 males) were included in the final analysis, with a median age of 56.70 years (interquartile range [IQR]: 50.70, 63.60) and a median disease duration of 84.0 months (IQR: 37.75, 132.00) (**Table 1**). Of these patients, 98 (42 females and 56 males) were positive for total IAs after insulin therapy, whereas 418 (211 females and 207 males) were negative for total IAs. As shown in **Table 1**, there were no statistical differences in terms of gender, age, diabetic duration, blood pressure, body mass index, blood lipid profile (LDLC, HDLC, TG, TC), hepatorenal function, hemoglobin, erythrocyte, leukocyte, and thrombocyte between the two groups before insulin therapy. Moreover, there were no significant differences in fasting plasma glucose, 2-hour postprandial blood glucose, HbA1c, daily insulin dosage, and fasting insulin. Serum direct bilirubin (3.85 [IQR: 2.69, 5.09] vs. 3.40 [IQR: 2.38, 4.40]; P = 0.016) was slightly elevated in the IA-positive group at baseline compared to the control group.

**Table 1:** Clinical and demographic characteristics of the study population beforeinsulin therapy

Characteristic	Total (n=516)	IA-negative (n=418)	IA-positive (n=98)	P value
Male (%)	263 (50.97%)	207 (49.50%)	56 (57.10%)	0.174
Age at enrollment (years)	56.70 (50.70 - 63.60)	56.70 (50.70- 63.60)	57.70 (49.90 - 63.70)	0.838
Duration of diabetes (months)	84.00 (37.75 - 132.00)	84.00 (36.00 - 132.00)	95.5 (59.00 - 132.00)	0.217
Systolic Blood Pressure (mmHg)	126.66 ± 14.45	126.69 ± 14.42	126.55 ± 14.65	0.932
Diastolic Blood Pressure (mmHg)	79.38 ± 9.43	79.39 ± 9.44	79.34 ± 9.41	0.962
BMI (kg/m^2^)	25.66 ± 3.19	25.71 ± 3.23	25.50 ± 3.05	0.561
HbA1c (%)	8.60 (7.90 - 9.80)	8.70 (7.90 - 9.80)	8.50 (7.88 - 9.0)	
HbA1c (mmol/mol)	70.49 (62.84 - 83.61)	71.58 (62.84 - 83.61)	69.40 (62.62 - 82.51)	0.603
Daily insulin dosage (unit per kilogram)	0.29 (0.23 - 0.36)	0.29 (0.22 - 0.35)	0.30 (0.24 - 0.37)	0.143
FPG (mmol/L)	10.71 (8.80 - 13.67)	10.70 (8.64 -13.62)	10.79 (9.29 -13.83)	0.443
2hPG (mmol/L)	17.70 (15.09 - 20.91)	17.44 (15.10 - 20.91)	17.94 (14.97 - 20.90)	0.716
Fasting insulin (uU/mL)^*^	10.30 (7.00 - 15.05)	9.64 (6.67 - 14.75)	11.00 (7.99 - 16.38)	0.059
Total cholesterol (mmol/L)	4.77 ± 1.16	4.78 ± 1.17	4.71 ± 1.09	0.576
Triglyceride (mmol/L)	1.64 (1.14 - 2.37)	1.68 (1.14 - 2.37)	1.48 (1.13 - 2.46)	0.610
HDLC(mmol/L)	1.150 (0.99 -1.36)	1.15 (0.99 - 1.36)	1.16 (0.99 - 1.37)	0.884
LDLC (mmol/L)	2.77 ± 0.87	2.78 ± 0.88	2.72 ± 0.81	0.571
ALT (U/L)	21.00 (15.00 - 32.00)	21.00 (15.00 - 31.00)	22.00 (14.93 - 32.25)	0.375
AST (U/L)	19.80 (16.00 - 25.00)	19.00 (16.00 - 25.00)	20.20 (16.00 - 27.28)	0.240
TBil (umol/L)	13.10 (10.20 - 16.30)	13.10 (10.19 - 16.10)	13.50 (10.25 - 17.15)	0.407
DBil (umol/L)	3.50 (2.40 - 4.60)	3.40 (2.38 - 4.40)	3.85 (2.69 - 5.09)	0.016
Blood urea nitrogen (mmol/L)	5.29 ± 1.38	5.29 ± 1.41	5.33 ± 1.24	0.785
Serum creatinine (umol/L)	59.24 ± 15.82	58.75 ± 15.63	61.36 ± 16.56	0.142
Hemoglobin (g/L)	143.6 ± 14.43	143.25 ± 14.37	145.04 ± 14.67	0.278
Erythrocyte (10^12/L)	4.77 ± 0.48	4.77 ± 0.48	4.79 ± 0.47	0.679
Leukocyte (10^9/L)	6.61 ± 1.51	6.58 ± 1.56	6.76 ± 1.31	0.283
Thrombocyte (10^9/L)	229.3 ± 56.01	228.66 ± 56.84	232.09 ± 52.52	0.585

Data for continuous variables are expressed as the mean±SD, or as the median (interquartile range). Categorical variables are presented as n (%). BMI, body mass index; FPG, fasting plasma glucose; 2hPG, 2-hour postprandial blood glucose; HbA1c, glycated hemoglobin; LDLC, low-density lipoprotein cholesterol; HDLC, high-density lipoprotein cholesterol; ALT, alanine aminotransferase; AST, aspartate aminotransferase; TBil, total bilirubin; DBil, direct bilirubin. * means that data of serum insulin before insulin administration were available in 95 IA-positive patients and 416 IA-negative patients.

### Insulin-treated T2D patients with IAs predominantly responded to IgG, followed by IgE

**Figure 1:**
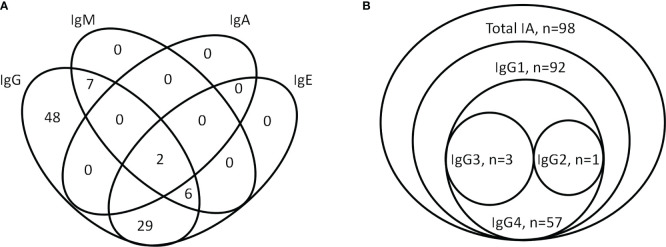
Venn diagram showing the number of individuals tested positive for subclass-specific insulin antibodies (IgG, IgA, IgE, and IgM) **(A)** and isotype-specific IgG-IA (IgG1-4) and total IA **(B)**. IA, insulin antibody.

**Figure 2:**
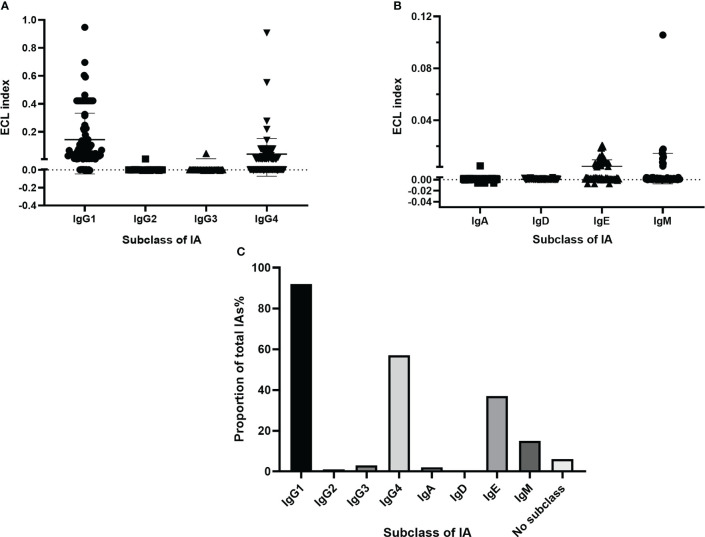
Levels (ECL index) of IgG-IA (IgG1-4) **(A)** and IgA-IA, IgD-IA, IgE-IA, IgM-IA **(B)**, and frequencies of positivity of different IA isotypes in patients detected positive for total IAs **(C)**. The solid lines represent median values, and the dotted horizontal lines denote the threshold for positivity. ECL, electrochemiluminescence; IA, insulin antibody.

Of the 98 study patients who were detected to be positive for total IAs, 92 had subclass IAs as follows: 48 (48.98%) had IgG-IA only; 29 (29.59%) had IgG-IA plus IgE-IA; 7 (7.14%) had IgG-IA plus IgM-IA; 6 (6.12%) had IgG-IA, IgE-IA, and IgM-IA; and 2 (2.04%) had IgG-IA plus IgA-IA, IgE-IA, and IgM-IA (**Figure 1A**), and thier ECL indexes are shown in **Figures 2A, B**. However, subclasses of IAs were not measurable in 6 patients, and IgD-IA was absent in all patients. Of the 92 subjects with the IgG-IA subclass, all were detected to have IgG1-IA, and 57 cases had IgG4-IA, whereas IgG2-IA and IgG3-IA were found in 1 and 3 cases, respectively (**Figures 1B**, **2C**).

### IAs associated with serum total insulin increase but not glycemic control

**Figure 3:**
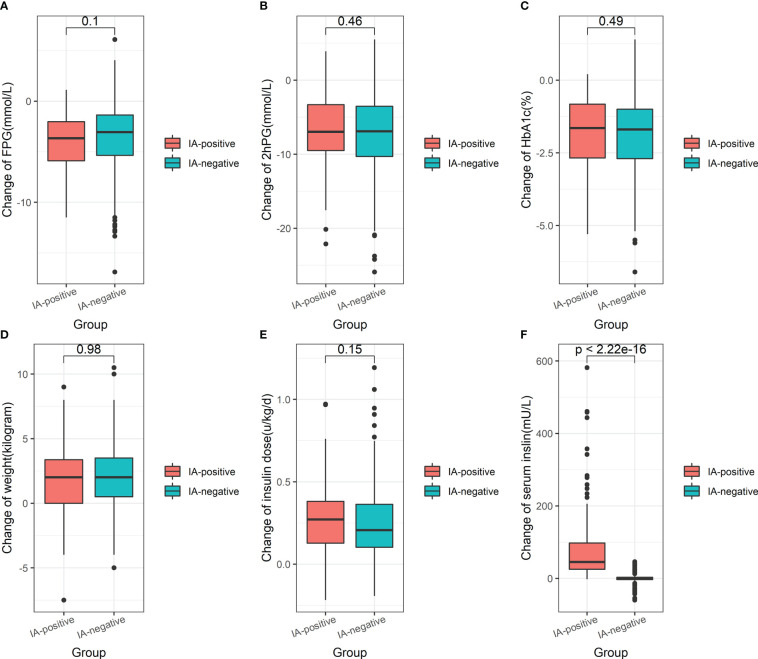
The boxplot showing the median change of FPG **(A)**, 2hPG **(B)**, HbA1c **(C)**, weight **(D)**, serum total insulin **(E)**, and daily insulin dose **(F)** from baseline in IA-positive patients and IA-negative patients. FPG, fasting plasma glucose; 2hPG, 2-hour postprandial blood glucose; HbA1c, glycated hemoglobin. Data on serum fasting insulin measured before and post-insulin administration were available in 94 IA-positive and 407 IA-negative patients.

Glycemic control, as indicated by the median change from baseline in FPG, 2hPG, and HbA1c, did not differ between the IA-positive group and the placebo group (-3.7 mmol/L [IQR: -5.9, -2.0] vs. -3.1 mmol/L [IQR: -5.4, -1.4], P = 0.10; -7.0 mmol/L [IQR: -9.5, -3.3] vs. -6.9 mmol/L [IQR: -10.3, -3.5], P = 0.46; -1.7% [IQR: -2.7, -0.8] vs. -1.7% [IQR: -2.7, -1.0], P = 0.49; **Figures 3A–C**). There was also no significant difference in weight change (2.0 kg [IQR: 0.0, 3.4] vs. 2.0 kg [IQR: 0.5, 3.5], P = 0.98; **Figure 3D**) between the two groups of patients before and after insulin treatment. IA-positive patients received approximately 900 times more serum insulin changes compared with IA-negative patients (45.0 uU/ml [IQR: 25.3, 97.7] vs. 0.05 uU/ml [IQR: -3.4, 3.1], P < 0.0001; **Figure 3E**), but the increase in daily insulin requirement over our observation period was similar between the IA-positive group and the IA-negative group (0.27 U per kg per day [IQR: 0.13, 0.38] vs. 0.21 U per kg per day [IQR: 0.10, 0.36], P = 0.15; **Figure 3F**).

### IgE-IA and IA subclass numbers associated with increased serum total insulin level

**Figure 4:**
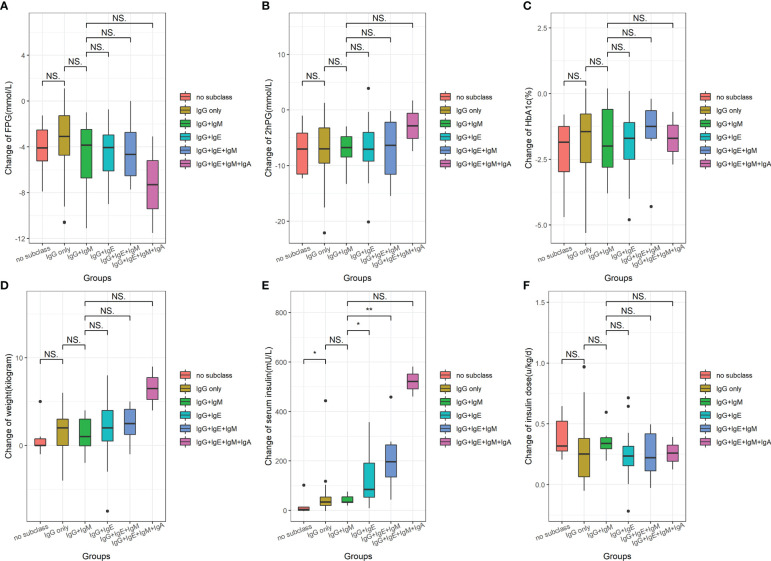
The boxplot showing the median change of FPG **(A)**, 2hPG **(B)**, HbA1c **(C)**, weight **(D)**, serum total insulin **(E)**, and daily insulin dose **(F)** from baseline among different IA subclasses groups. NS, no significance; * P<0.05; ** P<0.005. FPG, fasting plasma glucose; 2hPG, 2-hour postprandial blood glucose; HbA1c, glycated hemoglobin. Data of serum fasting insulin measured prior to and post-insulin administration were available in 94 IA-positive patients.

Among patients with different IA subclasses, alterations in FPG, 2hPG, HbA1c, weight, and daily insulin dose were similar (**Figures 4A–D, F**) before and after insulin treatment. A few minor differences remained—i.e., more decreased FPG (**Figure 4A**), less decreased 2hPG (**Figure 4B**), and more increased weight (**Figure 4D**)—and were observed in patients with all four subtypes (IgG-IA, IgE-IA, IgA-IA, and IgM-IA) compared to those in other groups, albeit not significantly. Patients with IgG-IA and IgE-IA, whether containing other subclasses or not, all had higher serum insulin than those with IgG-IA only or patients with IgG-IA and IgM-IA (**Figure 4E**), showing an increasing trend with the increase of IA subclass numbers.

### IAs not associated with hypoglycemia but with injection-site reactions

**Figure 5:**
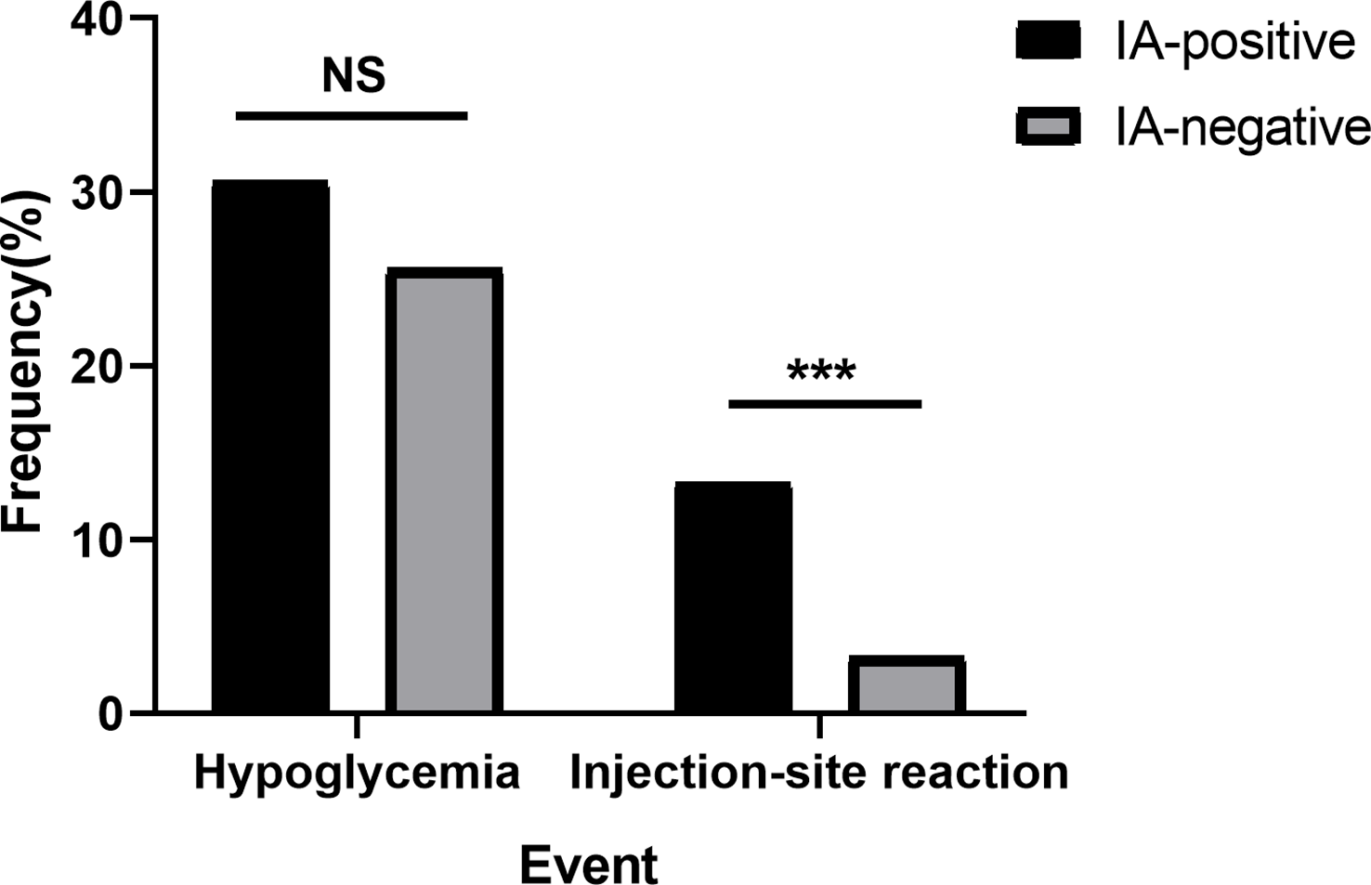
Frequency of hypoglycemia or injection-site reactions in the IA-positive group and IA-negative group. NS, no significance; *** P < 0.0001; injection-site reaction, referring to skin itching, local redness and swelling, ecchymosis, subcutaneous nodules, and urticaria.

Regarding insulin-associated adverse events, the injection-site reaction incidence was about four times higher in the IA-positive group than in the IA-negative group (**Figure 5**; 13.3% vs. 3.3%, P < 0.0001). However, the frequency of hypoglycemia was slightly higher in the IA-positive group than in the control group (**Figure 5**; 30.6% vs. 25.60%, P = 0.312).

### IgE-IA correlated more strongly with local response and IgM-IA correlated more strongly with hypoglycemia

**Figure 6:**
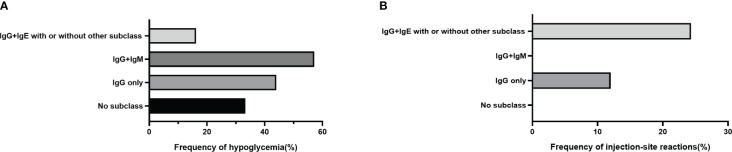
Frequency of hypoglycemia or injection-site reactions in the IA-positive group stratified by IA subclasses. **(A)** shows 2 of 6 (33.33%), 18 of 48 (37.50%), 4 of 7 (57.14%), and 6 of 37 (16.21%) patients with no subclass, IgG-IA alone, IgG-IA and IgM-IA, or IgG-IA, IgE-IA whether with or without other subclasses had hypoglycemia respectively. **(B)** shows 4 of 48 (8.30%), and 9 of 37 (24.32%) patients with IgG-IA only, or IgG-IA, IgE-IA, whether with or without other subclasses had injection-site reactions, respectively; other groups had no local responses.

To analyze insulin-associated events among patients with different IA subclasses, we divided them into four subgroups. As **Figure 6** shows, patients with IgG-IA and IgE-IA, whether containing other subclasses or not, had the lowest frequency of hypoglycemia (**Figure 6A**; 16.21%, 6 of 37 cases) and the highest frequency of injection-site reactions (**Figure 6B**; 24.32%, 9 of 37 patients). However, patients with IgG-IA and IgM-IA had the highest prevalence of hypoglycemia (**Figure 6A**; 57.14%, 4 of 7 cases), while they did not have injection-site reactions (**Figure 6B**; 0.0%).

## Discussion

This study demonstrated that insulin antibodies were present in approximately 20% of T2D patients treated with premixed insulin analogs for short-term therapy. IgG-IA was the predominant subclass distribution of insulin antibodies (IgG1 was the most dominant isotype of IgG), followed by IgE-IA. However, IA subclasses were not detectable in six patients. One possible explanation is that some non-specific bindings were measurable in detecting the total insulin antibodies, leading to false positives for IAs. Another reason was that the relevant total insulin antibodies were genuinely positive, while the low indexes when detecting IA subclasses were reported as negative to ensure accuracy. Taken together, these factors indicate the test’s limitations, which comprise a common clinical testing phenomenon. A similar description was reported in the work of Martin Fuchtenbusch et al. (16), in which 2 of 12 patients failed to register any IA subclasses.

According to previous studies, conventional bovine–porcine insulin produces antibodies in > 95% of insulin-treated patients (21, 22). A study (23) examining the immunogenicity of different monocomponent insulins in newly diagnosed patients with type 1 diabetes has shown both human and porcine insulin groups had 24% and 39% of patients with IAs at three months. In another study of > 200 patients without previous exposure to insulin, 44% of patients taking human insulin developed insulin antibodies compared to 60% of those taking porcine insulin at 12 months (5). Accordingly, by using purified and recombinant human insulin preparations, IAs have been markedly reduced but not eliminated. IgG subclass responses to insulin may vary with diabetes type. For example, in T2D patients with high levels of insulin antibody responses, IgG1, IgG3, and IgG4 antibodies have been shown to be elevated, but IgG2 antibodies negligibly absent (24). This is similar to the distribution of IgG subclasses in our study. The frequency of IgG3-IA was lower in our patients. Notably, the IgG1 and IgG4 were the most common subclass responses to both insulin autoantibodies (IAAs) and IAs in patients with type 1 diabetes and insulin-treated prediabetic patients with islet antibody positivity (16). When genetically susceptible young children lack the IgG3-IA, they may be protected from type 1 diabetes (17); conversely, type 1 diabetic patients have been shown to have an elevated IgG3-IA.

**Figure 3** illustrates that there was no significant correlation between IAs and glucose control as reflected by FPG, 2hPG, or HbA1c, consistent with most studies from the 1980s and 1990s (6). Similarly, only a marginal effect on glucose control was observed for IAs induced by subcutaneous or peritoneal insulin infusions (25). Recently, Philip Home et al. (18) demonstrated no relationship between maximum individual IA titers and changes in HbA1c or insulin dose. However, in our study, more decreased FPG, less decreased 2hPG, and more increased weight were observed in patients with all four subtypes (IgG-IA, IgE-IA, IgA-IA, and IgM-IA), albeit not to the point of statistical significance. The possible explanation for the minor difference provided was that insulin antibodies in these patients with four IA subtypes might bind more tightly (high affinity) to insulin and delay its release, resulting in higher postprandial glucose and less decrease in 2hPG compared with other groups. Meanwhile, delayed hyperinsulinemia has been shown to result in lower fasting glucose and a greater decrease in FPG (26). Unfortunately, due to the limitations of retrospective studies, this remains speculation on our part and warrants confirmation by testing the affinity of different IA subclasses. Regarding insulin dosage requirements, the IA-positive group appeared to require slightly more insulin than the control group, despite there being no statistical difference, suggesting that IAs may not be associated with immune insulin resistance in the short-term, while the long-term effects must be determined by subsequent studies. Although it has been documented that patients with high IA levels may present with a rare syndrome of severe insulin resistance (requiring more than 200 U/d of insulin for at least two days) (9, 27), the underlying causal mechanism remains unclear. Additional prospective treatment trials involving human and animal insulin, insulin analogs, and inhaled insulin trials have also shown no significant correlation between IA levels and insulin dose in insulin-naive and insulin-treated patients (5, 7, 28).

Hypoglycemia is a common adverse effect of insulin therapy and an indicator that the safety of insulin requires evaluation. IAs have often been considered relevant to hypoglycemia, especially in EIAS (10). Nevertheless, in our observation, IAs had no relationship with hypoglycemia episodes but instead with increased total serum insulin. Similarly, Fineberg et al. (6) concluded that hypoglycemic events and IA levels were not correlated. Furthermore, sporadic case reports indicated that high levels of IA were associated with clinical hypoglycemia syndromes in a few individuals (29–32); however, none of these studies analyzed IA subtypes. Hypoglycemia in this setting was potentially caused by increased insulin dissociation from the insulin-antibody complex due to low affinity or decreased glucose counter-regulation and prolonged free insulin half-life (5, 33, 34), while other mechanisms warrant further investigation. Whether such low-affinity insulin antibodies are more likely to occur in patients with specific IA isoforms or isoform combinations also deserves further investigation. A previous study (35) on IAAs predicting type 1 diabetes showed that IgM antibodies were of lower affinity than IgG antibodies. Interestingly, in our subgroup analysis, patients with IgG-IA and IgM-IA were more susceptible to hypoglycemia than other subtype combinations; however, patients with IgG-IA and IgE-IA were not susceptible, on the contrary. We speculated that the pentameric structure of IgM-IA might have high capacity and low affinity—that is, it might bind more insulin (high capacity) than other IA subclasses, as well as being more easily dissociated (low affinity). The complex, which combined IgM-IA and insulin, dissociated, then more insulin was released, resulting in a higher incidence of hypoglycemia. The lowest frequency of hypoglycemia in patients with IgG-IA and IgE-IA might be related to the high affinity of IgE-IA; in this case, high-affinity IgE-IA is minimally dissociated, thus leading to less hypoglycemia. Additionally, it has been shown that high-affinity but not low-affinity IgE causes anaphylaxis (36, 37). The detection of IgG-IA, IgM-IA, and IgE-IA in patients with severe hypoglycemia might be suitable to test this hypothesis and provide more insight into the mechanisms involved. In addition, one possible explanation for our study’s discrepancy from the previous studies is that the latter mainly enrolled patients with recurrent hypoglycemia or severe hypoglycemia. In contrast, there were few cases of severe hypoglycemia in our present study. Another reason is that, despite elevated insulin levels in our patients with IAs, there may be no abnormal dissociation of insulin as previously described. Notably, hyperinsulinemia has been demonstrated to contribute to diastolic cardiovascular dysfunction and diabetic cardiomyopathy (38, 39). Investigating whether hyperinsulinemia caused by specific IA subtypes is more predictive of this risk or is associated with it will be useful.

In the past, allergy was another frequent adverse effect in patients receiving insulin therapy (40). The prevalence of insulin allergy has decreased since human insulin and its analogs were introduced (41). Such hypersensitivity may result from the insulin molecule itself, as well as from protamine and other components. However, in our study, IA-positive patients suffered a higher rate of injection-site reactions, indicating allergy but unsuitable for all cases, especially in subjects with IgE-IA. Immunoglobulin E is central to type I immediate allergic responses (42). In addition, insulin-specific IgE (type 1) and IgG (type 3) antibodies may mediate local and systemic reactions to insulin administration (6, 43). Additionally, a type IV response can also contribute to insulin hypersensitivity (44). Thus, it is easy to understand why more local reactions occurred in our patients with IgG-IA and IgE-IA. Moreover, IgE-IA has been demonstrated to be present in injection-site reactions by other researchers (45), as has IgG-IA, IgM-IA, and IgA-IA. However, the exact nature of these patients’ local responses has yet to be identified, and in-depth evaluations of such patients have not been performed (45).

Finally, it is essential to note that our study has several limitations. First, retrospective data from a single center are subject to the inherent limitations of such investigations. Second, the affinity of IAs or IA subclasses was not routinely performed in clinics. The affinity may indicate the maturity of the immune response (35), and such differences may explain why specific subtypes of IA are more prone to hypoglycemia and other related problems. Third, HLA genotypes have been shown to be associated with IA isotype and affinity (46). However, patients’ HLA genotypes were not available in this study, and thus a relationship between IA subclasses and genotypes was not explored. Moreover, the lack of follow-up data prevented us from evaluating the correlation between IA subclasses and long-term clinical outcomes and seroconversion among different IA subclasses. This deficiency will be explored in future work.

In summary, our findings indicated that IA subclasses might be correlated with adverse effects of premixed insulin analog therapy, despite showing no association with glycemic control. We offer a suggestion for clinical designers and clinicians: If patients exhibit unexplained hypoglycemia or other adverse reactions, IA subtypes should be considered in addition to testing for IAs. In future work, we desire to shed more light on the mechanisms responsible for the maturation of the immune responses to exogenous insulin. Another future study will focus on exploring the relationship between IA subclasses and long-term clinical outcomes.

## Data availability statement

The original contributions presented in the study are included in the article/**Supplementary Material**. Further inquiries can be directed to the corresponding authors.

## Ethics statement

This project was approved by the ethics committee of the First Affiliated Hospital of Nanjing Medical University (2021-SR-075). The patients/participants provided their written informed consent to participate in this study. Written informed consent was obtained from the individual(s) for the publication of any potentially identifiable images or data included in this article.

## Author contributions

SC, HC, and YJ contributed equally to the manuscript and shared the first authorship. XZ and MZ contributed to data collection. TY and YG designed the study and revised the manuscript. All authors contributed to the article and approved the submitted version.
