# Supplementary figures and images for: Association of subclass distribution of insulin antibody with glucose control in insulin-treated type 2 diabetes mellitus: a retrospective observational study
Source: Front Endocrinol (Lausanne). 2023 Apr 18;14:1141414. doi: 10.3389/fendo.2023.1141414 (PMC10151736; doi:10.3389/fendo.2023.1141414)

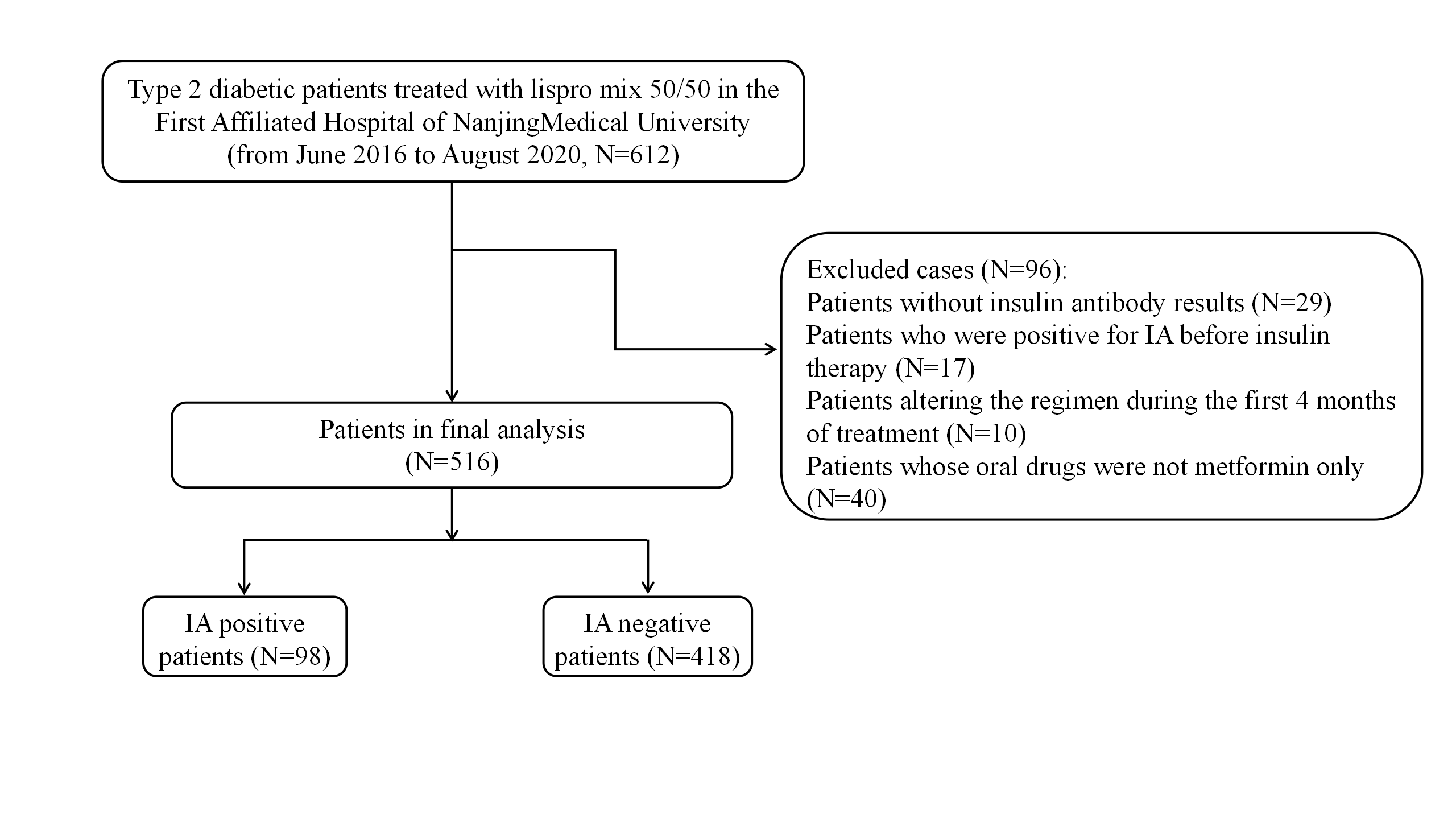

Supplement: Supplementary file 1 [file Image_1.tif]
